# Conflict Processing Is Unaffected by Stimulus Duration Across Multiple Visual Tasks: Evidence for Transient over Permanent Activation Models

**DOI:** 10.1007/s42113-024-00211-x

**Published:** 2024-07-24

**Authors:** Ruben Ellinghaus, Roman Liepelt, Ian G. Mackenzie, Victor Mittelstädt

**Affiliations:** 1 Department of General Psychology: Judgment, Decision Making, Action, Faculty of Psychology, FernUniversität in Hagen, Hagen, Germany; 2 Universität Tübingen, Tübingen, Germany

**Keywords:** Conflict tasks, Selective attention, Action selection, Diffusion model, Simon task, Flanker task, DMC, Delta plots

## Abstract

Permanent activation models of conflict tasks assume that the distractor constantly provides input into the response selection process as long as the stimulus is presented, whereas transient activation models assume that the influence of distractors is independent of stimulus duration. In the present study, we contrasted the prediction of these two architectures across different visual conflict tasks with manual responses. Specifically, we investigated the effects of short (150 ms) versus response-terminated stimulus duration on the delta plot (DP) slopes in a classic and accessory Simon task (Experiment 1), in a classic Simon and Eriksen flanker task (Experiment 2), and in a word-based (semantic) Simon and classic Stroop task (Experiment 3). Contrary to permanent activation models, all experiments revealed that the DP slopes were largely unaffected by stimulus duration. This result pattern is consistent with transient activation models such as the diffusion model for conflict tasks (DMC). Coherently, DMC was successfully fitted to the data. Importantly, the $\tau$ parameter reflecting the time course of distractor activation, as well as other parameters, were largely unaffected by stimulus duration. In conclusion, our results suggest that distractor activation in visual conflict tasks with manual responses is transient rather than permanent, and stimulus duration generally has no meaningful influence on conflict processing.

## Introduction

Humans and other agents act in a goal-directed manner. As a prerequisite, they must select relevant information but at the same time discard distracting information from further processing. A major intent of cognitive psychology is to better understand the processing mechanisms underlying the selection and transformation of sensory information into motor responses (Balota et al., 2008; Donders, 1969; Ellinghaus & Miller, 2018; Luce et al., 1986; Luo & Proctor, 2018; Mittelstädt & Miller, 2020; Sternberg, 1969; Townsend et al., 1983). The present study aims to contribute to this goal by investigating whether and how the duration of presenting information in conflict tasks affects processing or not. Specifically, we employ distributional analyses alongside model-based analyses to particularly examine whether distracting information has a permanent impact on processing during various conflict tasks or whether it should be conceptualized as part of a transient processing architecture.

### Conflict Tasks

In conflict tasks, participants are instructed to react as fast and as accurately as possible to some target information source, while a distracting information source is also present. For instance, in the visual variant of the classic Simon task (Simon, 1969; reviews given by Hommel, 2011; Lu & Proctor, 1995), participants indicate by left and right button presses the identity of a stimulus such as a letter (target information). This letter is presented either in the left or in the right field of the screen (location $=$ distracting information). In the Eriksen flanker task (Eriksen & Eriksen, 1974; for a review see Eriksen, 1995), participants respond to the identity of a central target flanked by distractors that indicate the same or a different response (e.g., HHH versus SHS). As a general finding across these tasks, mean reaction times (RTs) and mean error rates (ERs) are typically higher in trials when the target and distractor are associated with different responses (incompatible condition) as compared to when they are associated with the same response (compatible condition), indicating that task-irrelevant distractor activation cannot be fully prevented from influencing target-based processing.

In order to account for this phenomenon, most cognitive architectures proposed to underlie performance in conflict tasks rest on the distinction between controlled and automatic processing (Norman & Shallice, 1986; Posner et al., 2004; Schneider & Shiffrin, 1977). Specifically, these accounts assume that the target and distractor information in conflict tasks are respectively processed in an intentional (i.e., controlled) and a reflex-like (i.e., automatic) manner (e.g., Cohen et al., 1990; Coles et al., 1985; Hommel, 1993; Logan, 1980; Ridderinkhof et al., 2002). For example, the response tendency activated by the distractor might spill over into the controlled response selection process in a synergistic or antagonistic manner, respectively speeding or slowing down response selection in compatible and incompatible trials (Ulrich et al., 2015).

In addition to comparing mean RT and mean errors between conditions, it may sometimes be interesting to also investigate the time course of an experimental effect. For example, delta plots (DPs) plot the difference between compatible and incompatible mean response times (RTs) separately at RT percentiles ranging from the fastest to the slowest RTs (e.g., 10%, 20%, ..., 90%). For most conflict tasks, the DP slopes are primarily reported to be increasing, indicating that relatively slow responses are affected more strongly by the conflicting information than faster responses. This has been documented for the Eriksen flanker task, the Stroop task, and vertical variants of the Simon task (Burle et al., 2014; e.g., Proctor et al., 2011; Ridderinkhof et al., 2005). For the classic Simon task, mainly decreasing delta plots have been reported (De Jong et al., 1994; Pratte et al., 2010; Servant et al., 2014; Vallesi et al., 2005; Wiegand & Wascher, 2005).

### Permanent Versus Transient Distractor Activation

As explained in more detail next, DPs might be useful in discriminating between two classes of dual-route models, namely transient and permanent activation models. Specifically, permanent activation models assume the distractor to constantly input into the automatic process as long as the stimulus is presented, implying activation to monotonously increase with stimulus duration (e.g., Cohen et al., 1990; Logan, 1980; cf. McClelland, 1979). Alternatively, transient activation models assume that the influence of the automatic activation lessens during the course of a trial (Ulrich et al., 2015; Usher & McClelland, 2001; Wühr & Heuer, 2017; Wühr & Heuer, 2018; Zorzi & Umiltá, 1995). For example, the diffusion model for conflict tasks (DMC, Ulrich et al., 2015) assumes that distractor activation is brief and pulse-like, increasing after stimulus onset and fading after reaching its peak due to passive decay (Hommel, 1993, 1994) or active inhibition (Neill & Westberry, 1987; Ridderinkhof et al., 2002; Ridderinkhof, 2002). Critically, permanent and transient activation models make different predictions regarding DP slopes as a function of stimulus duration (Ellinghaus et al., 2018). Assuming that automatic activation is transient, the slope of the DPs may be virtually independent of stimulus duration. Permanent activation models, on the other hand, imply that with a short stimulus duration, the distractor has less time to exert its influence, which should lead to increasingly positive DPs with increasing stimulus duration.

While there is much research showing that controlled processing is independent of stimulus duration in non-conflict tasks (for an overview, see Ratcliff et al., 2016), the influence of stimulus duration on conflict processing has been rarely investigated. In fact, we are only aware of the studies by Xiong and Proctor (2016) and Ellinghaus et al. (2018) that manipulated stimulus duration experimentally, respectively in an auditory and visual Simon task. Both studies observed that the negative DP slopes were unaffected by the duration manipulation. Thus, these studies suggest a transient rather than permanent distractor activation in the Simon task. From a theoretical perspective, however, it is important to clarify whether transient activation architectures also underlay other conflict tasks, as postulated by many prominent models (Hübner et al., 2010; Ulrich et al., 2015; Wühr & Heuer, 2017, 2018). This generalization seemed a priori questionable and therefore required empirical investigation. For example, it might be that only direct motor activation is transient over time, and this type of activation is primarily involved in the classic Simon task (Stürmer et al., 2002; Valle-Inclán, 1996; Valle-Inclán & Redondo, 1998), while other conflict tasks might also require a more conceptual analysis or higher-level code interference (Wascher et al., 2001; Wiegand & Wascher, 2005).

### Present Study

Accordingly, the goal of the present study was to test the hypothesis that stimulus duration influences the time course of compatibility effects, as predicted by permanent activation models, across a range of different conflict tasks. To achieve this aim, we conducted three experiments where we investigated the influence of stimulus duration (150 ms versus response-terminated) on delta plot slopes across multiple visual conflict tasks with manual responses. That is, we compared the classic Simon versus the accessory Simon (Experiment 1), the classic Simon versus the classic flanker task (Experiment 2), as well as the word-based Simon versus the classic Stroop task (Experiment 3). These tasks were chosen because they differ from each other in terms of delta plots (i.e., primarily negative delta plots in the classic Simon task, but flat or increasing delta plots in the other tasks employed) and also, at least partially, in the type of conflict. For example, in the classic Simon task, spatial location primarily produces Stimulus-Response (S-R) conflict (Kornblum et al., 1990). While this type of conflict may also be present in the other conflict tasks employed in the current study, there might be additional perceptual-based Stimulus-Stimulus (S-S) conflict in the Eriksen flanker and Stroop tasks (Kornblum et al., 1990; Scerrati et al., 2017) or semantic code interference in the word-based Simon and Stroop tasks (Goldfarb & Henik, 2007). Thus, not withstanding obvious similarities between these experimental paradigms, there are also potential differences regarding the mechanisms underlying conflict processing in these tasks, as is also supported by empirical studies that demonstrate task-specific responses to certain experimental manipulations (Mackenzie et al., 2022; Mittelstädt et al., 2023a, b; Scerrati et al., 2017).

Thus, with this set of experiments, we can test the influence of stimulus duration (and, with that, the predictions of permanent versus transient models) across a broad range of visual conflict tasks. Moreover, we aimed to compare these tasks in additional model-based analyses, as explained in the corresponding section of the paper in more detail.^1^

## Experiment 1

In Experiment 1, we investigated the effects of stimulus duration (short versus response-terminated) on delta plot slopes in a classic and an accessory Simon task. The accessory Simon task was chosen because it is structurally similar to the classic variant but tends to produce increasing rather than decreasing delta plots (Magen, 2019; Proctor et al., 2005). In case such an increase of the compatibility effect across time is the result of a permanent distractor-based activation, the delta plots should be less positive (or even negative) for the short duration compared to the long duration. At the same time, the absence of an effect of duration would favor transient models underlying the task. Regarding the classic Simon task, we expected no effect of duration on DP slopes - thus replicating the null-effect reported by Ellinghaus et al. (2018) - constituting a further challenge for models assuming permanent distractor activation for this task. In essence, this experiment allowed us to investigate whether the distinction between permanent and transient distractor-based activation models would generalize across tasks that primarily differ in the time course of distractor-based activation rather than the type of conflict (i.e., primarily S-R conflict in both cases).

### Methods

#### Participants

Eighty participants (51 women, with a mean age of 42.4 years (SD $=$ 12.01) remained for analysis (an additional 6 participants were excluded because of error rates of 20% or higher).^2^ They were undergraduates from the University of Hagen and received course credit for participation.

#### Sample Size Justification

The preregistered sample size was determined by both practical constraints (e.g., participant availability and resources) and empirical constraints (e.g., effect sizes in previous studies), as well as theoretical considerations. Specifically, previous delta plot studies typically observed, if present, rather large differences between delta plot slopes (Mackenzie et al., 2022). However, we decided to test a relatively larger sample size because: a) detecting potentially smaller effects seemed still theoretically meaningful to us b) a previous study using the classic Simon task observed no effect with a similar manipulation (Ellinghaus et al., 2018) and c) we also wanted to be sufficiently powered for potential differences as a function of the specific conflict type. For example, with a sample size of 80 participants, we would have a statistical power of 1 − $\beta$ $=$ 0.80 (using a significance level of $\alpha$ $=$ 0.05) to detect a significant difference in delta plot slopes in the paired t-test of at least $d_z$ $=$ 0.27 (one-sided), or to detect a significant interaction in delta plot slopes of at least $\eta _{p}^{2}$ $=$ 0.10 in the 2x2 ANOVAs. Considering that we complemented our empirical delta plot analyses with additional modeling-based analyses, we considered the planned sample size of 80 participants in each experiment sufficient to draw meaningful conclusions regarding our question in terms of permanent vs. transient distractor-based activations.

#### Stimuli and Apparatus

The experiment was conducted online using the JavaScript library jsPsych (De Leeuw, 2015), hosted on Pavlovia (cf. Bridges et al., 2020). A link was made available to participants enabling them to complete the experiments with their personal computers. At the start of the experiment, participants were asked to adjust the size of a rectangle until it matched the dimensions of a credit card. This calibration routine ensured approximately equal stimulus size across participants.

Stimuli were presented in black on a gray background. A plus sign served as a fixation cross. Target stimuli consisted of the letters S and H. Responses were respectively given with left and right index fingers on the Q and P buttons of a German QWERTZ keyboard. The S-R mapping was randomly selected for each participant.

#### Task and Procedure

In classic Simon (CS) task blocks, the target letter appeared to the left or right of the center of the screen. In accessory Simon (AS) task blocks, the target letter was centrally presented and an additional letter of the same identity was presented to the left or right.

Task (CS vs. AS) were held constant within a block and alternated across blocks (e.g., block 1: CS, block 2: AS, block 3: CS, block 4: AS,..). Half of the participants started with a CS block and the other half with an AS block. Stimulus duration varied (short, long) randomly within a block. Each of the 16 blocks consisted of 56 randomly ordered trials with 7 presentations of each of the eight possible stimulus displays (2 targets X 2 locations X 2 stimulus durations). Instructional screens at the beginning of each block served as a reminder of the stimulus-response mapping and upcoming tasks. After each block, participants could take a self-paced break and receive performance feedback (mean reaction time and percentage of errors).

In each trial, the fixation cross appeared on the screen for 500 ms, and following the offset of the fixation cross, a target letter was presented to the left or right (i.e., CS) or a target letter flanked by another letter to the left or right (i.e., AS) blocks. The duration of the stimuli in the long and short conditions was modeled after Ellinghaus et al. (2018). That is, in short-duration trials, the stimulus display was replaced by a black screen 150 ms after onset. In long-duration trials, the stimulus display remained on the screen until participants responded. Note that in both short and long duration, there was a response deadline of 1500 ms (starting from target onset). In case of an error or too slow/too fast responses, participants received feedback for 2000 ms stating whether (a) there was an error, (b) the response was too fast (< 150 ms), or (c) too slow (> 1500 ms). The next trial started after an interval of 750 ms.^3^

**Fig. 1:**
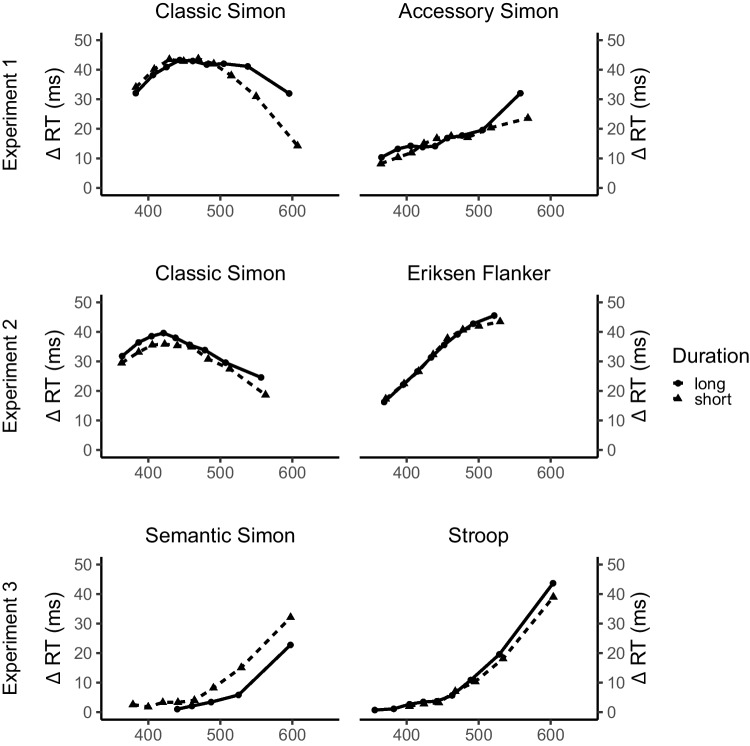
Delta Plots (i.e., the net mean effect within each decile plotted against the mean of the decile) as a function of *Duration* and *Task*. The upper, middle, and lower panels respectively depict Experiment 1, Experiment 2, and Experiment 3

#### Data Analysis

Only correct trials between 150 ms (anticipation cutoff) and 1500 ms (RT deadline) latencies were included for reaction time (RT) analysis. For DP construction, percentiles (10, 20, ..., 90%) were computed for each participant and condition separately. The corresponding slopes of both RT and ER slopes were estimated with a linear fit and then submitted to a two-factorial ANOVA with the factors *Duration* and *Task* (cf., Ellinghaus & Miller, 2018; Mittelstädt & Miller, 2018; Pratte et al., 2010).^4^ The results of the mean RT and ER analysis are given in the Appendix. For these analyses, RTs and ERs were submitted to a three-factorial ANOVA with the factors *Compatibility*, *Duration*, and *Task*.

### Results

As can be seen in the upper panel of Fig. 1, the DPs of the classic Simon task follow the typical negative-going trend, $$t(79) = -1.71$$, $$p =.045$$, $$d_z$$ = $$-$$0.19, while the net RT effect of the accessory Simon task increases across bins, $$t(79) = 5.12$$, $$p <.001$$, $$d_z$$ = 0.57. This qualitative difference between the time courses of the tasks was reflected in the significant main effect of *Task*, $$F(1, 79) = 22.90$$, $$ MSE = 0.05$$, $$p <.001$$, $$\hat{\eta }^2_p =.225$$. Furthermore, the main effect of *Duration* was significant, $$F(1, 79) = 8.80$$, $$ MSE = 0.03$$, $$p =.004$$, $$\hat{\eta }^2_p =.100$$, reflecting steeper slopes with the long as compared to the short stimulus Duration. Importantly, this effect of *Duration* was further modulated by *Task*, $$F(1, 79) = 4.79$$, $$ MSE = 0.02$$, $$p =.032$$, $$\hat{\eta }^2_p =.057$$; *Duration* had a significant influence on the slopes for classic Simon task, $$F(1, 79) = 16.87$$, $$ MSE = 0.02$$, $$p <.001$$, $$\hat{\eta }^2_p =.176$$, but not for the accessory Simon task, $$F(1, 79) = 0.50$$, $$ MSE = 0.03$$, $$p =.483$$, $$\hat{\eta }^2_p =.006$$. Moreover, for the classic Simon the slope was significantly below zero only for the short duration, $$t(79) = -3.95$$, $$p <.001$$, $$d_z$$ = $$-$$0.19 but not significantly different from zero for the long duration, $$t(79) = 0.42$$, $$p =.661$$, $$d_z$$ = $$-$$0.19.

### Discussion

The results of Experiment 1 are partially inconsistent with permanent activation models, but consistent with a transient activation account. Specifically, in the accessory Simon task, positive delta plots were observed for both duration conditions, and there was no evidence that the slope meaningfully differed across duration conditions. This result pattern poses a challenge for permanent activation models, as these models would predict the slopes to decrease with decreasing stimulus duration. However, for the classic Simon task, negative DPs were observed but the nDP declined with a steeper slope for the short duration than for the long duration. In fact, the slopes were reliably negative only for the short but not for the long condition. While this result pattern is predicted by permanent activation models, it should be noted that the effect was small and previously not observed by Ellinghaus et al. (2018). Therefore it is possible that the observed differences across studies are due to statistical reasons (i.e., Type 1 or Type 2 error).

## Experiment 2

Experiment 2 investigated the effects of stimulus duration on delta plot slopes in another prominent conflict task, the Eriksen flanker task. Although this task typically produces increasing delta plots, model-based research suggests that the flanker effect is based on the same transient interference mechanism as the Simon task (Luo & Proctor, 2020; Ulrich et al., 2015). Henceforth, observing that the flanker delta plots are not modulated by stimulus duration would lend further support for this view of a common underlying mechanism. On the contrary, under the presumption of a permanent activation architecture, the flanker delta plots should be less positive (or even negative) in the short-duration condition. At the same time, we conducted another test of the classic Simon task to address the partially inconsistent findings between Experiment 1 and an earlier study (Ellinghaus et al., 2018).

### Methods

#### Participants

81 participants (64 women, with a mean age of 33.2 years, SD $=$ 14.55) remained for analysis (an additional 8 participants were excluded because of error rates of 20% or higher). They were undergraduates from the University of Hagen and received course credit for participation.

#### Stimuli and Apparatus

Stimuli and apparatus were identical to Experiment 1.

#### Task and Procedure

The procedure was identical to Experiment 1, except that the accessory Simon task was replaced by an Eriksen Flanker task, wherein which the target was flanked by two distractors and presented centrally. Thus, in congruent trials of the Flanker task, both distractors and targets match (e.g., HHH), whereas, in incongruent trials, they mismatch (e.g., HSH).

#### Data Analysis

DP analysis was identical to Experiment 1, and mean RT and mean ER results are again given in the appendix.

### Results

As can be seen in the middle panel of Fig. 1, the DPs of the Simon task follow the typical negative-going trend, $$t(80) = -2.21$$, $$p =.015$$, $$d_z$$ = $$-$$0.25, while the net RT effect of the Flanker task increases across bins, $$t(80) = 11.79$$, $$p <.001$$, $$d_z$$ = 1.31. This qualitative difference between the time courses of the tasks was reflected in the significant main effect of *Task*, $$F(1, 80) = 70.07$$, $$ MSE = 0.06$$, $$p <.001$$, $$\hat{\eta }^2_p =.467$$. Most importantly, the main effect of *Duration* was not significant, $$F(1, 80) = 1.34$$, $$ MSE = 0.03$$, $$p =.250$$, $$\hat{\eta }^2_p =.017$$, reflecting no meaningful difference in slopes between the long and the short stimulus duration. Also, the effect of stimulus duration on the slopes did not meaningfully differ between the tasks, as indicated by the insignificant interaction of *Duration* and *Task*, $$F(1, 80) = 0.51$$, $$ MSE = 0.02$$, $$p =.476$$, $$\hat{\eta }^2_p =.006$$.

### Discussion

The results of Experiment 2 provided further inconsistencies with the predictions of permanent activation accounts. Rather, the results are consistent with transient architectures. In detail, neither the typical decreasing DPs for the Simon task nor the typical increasing DPs for the flanker task were modulated by the duration of the stimulus. Furthermore, the effect of stimulus duration on DP slopes in the classic Simon task which was observed in Experiment 1 was not replicated in Experiment 2. Since previous studies (Ellinghaus et al., 2018; Xiong & Proctor, 2016) did also not observe an effect of stimulus duration on the time course of the Simon effect, the finding of Experiment 1 appears to constitute an anomaly reflecting a tiny and/or unreliable effect. In sum, the results of Experiment 2 support the conclusion of Experiment 1 that distractor activation in conflict tasks is transient rather than permanent.

## Experiment 3

Experiment 3 investigated the effects of stimulus duration on delta plot slopes in a word-based (semantic) Simon task (e.g., identifying the font color of the written word “LEFT”) and a classic Stroop task (e.g., identifying the font color of the written word “GREEN”). Usually increasing delta plots have been reported for both the word-based Simon (e.g., Luo & Proctor, 2017; Pellicano et al., 2009) and the Stroop task (e.g., Kinoshita et al., 2017; Mittelstädt et al., 2023b; Pratte et al., 2010), suggesting that response activation by the irrelevant semantic information might not dissipate but strengthen over time. Notably, some of the classic permanent activation architectures were specifically modeled to explain behavioral performance in the Stroop task and related semantic tasks (e.g., Cohen et al., 1990; Logan, 1980). Coherently, semantic congruence effects in word-based priming tasks have been shown to increase with increasing prime duration, which is consistent with a conceptual stimulus-analysis constantly inputting into response activation (e.g., Smith et al., 1994). These considerations suggest that the delta plots observed in the two semantic tasks of Experiment 3 might be less increasing (or even decreasing) in the short-duration condition compared to the long-duration condition across both tasks. On the contrary, the absence of an effect of duration would again challenge permanent activation models but cohere with transient activation architectures such as DMC.

### Methods

#### Participants

81 participants (57 women, with a mean age of 34.0 years, SD $=$ 12.18) remained for analysis (an additional 5 participants were excluded because of error rates of 20% or higher). They were undergraduates from the University of Hagen and received course credit for participation.

#### Stimuli and Apparatus

Stimuli and apparatus were identical to Experiment 1.

#### Task and Procedure

The procedure of this experiment was again similar to the previous experiments, except for the following changes. Specifically, a semantic visual version of the manual Simon task was compared to a classic visual variant of the manual Stroop task. Thus, in this experiment, the target dimension was the font color (green or blue) of written words. The meaning of the words served as distractors and were the words “LEFT” or “RIGHT” for the semantic Simon task and “GREEN” and “BLUE” for the Stroop task written in German.

#### Data Analysis

DP analysis was identical to Experiment 1, and mean RT and mean ER results are again given in the appendix.

### Results

As can be seen in the lower panel of Fig. 1, the DPs of the Simon and Stroop task increase across bins, $$t(80) = 3.74$$, $$p <.001$$, $$d_z$$ = 0.42 and $$t(80) = 6.46$$, $$p <.001$$, $$d_z$$ = 0.72, respectively. The slopes did not meaningfully differ between the tasks, $$F(1, 80) = 3.70$$, $$ MSE = 0.06$$, $$p =.058$$, $$\hat{\eta }^2_p =.044$$. Most importantly, the main effect of *Duration* was not significant, $$F(1, 80) = 0.03$$, $$ MSE = 0.03$$, $$p =.874$$, $$\hat{\eta }^2_p =.000$$, reflecting no meaningful difference in slopes between the long and the short stimulus duration. Also, the effect of stimulus duration on the slopes did not meaningfully differ between the tasks, as indicated by the insignificant interaction of *Duration* and *Task*, $$F(1, 80) = 0.62$$, $$ MSE = 0.03$$, $$p =.435$$, $$\hat{\eta }^2_p =.008$$.

### Discussion

The results of Experiment 3 generalize the findings from Experiment 2 to two other conflict tasks (Stroop task and semantic Simon task) and hence constitute a further challenge for permanent activation accounts. Specifically, there was no evidence that the pDPs observed with the word-based Simon and classic Stroop tasks differed as a function of stimulus duration, as would have been predicted by permanent activation accounts.

## Diffusion Model for Conflict Tasks (DMC)

After establishing that the delta plot patterns in all three experiments were largely in line with the predictions of transient activation accounts, we also fitted the data to the diffusion model for conflict (DMC) and compared the best-fitting parameters as a function of our stimulus duration manipulation. DMC proposes that both controlled target-based activation and automatic distractor-based activation contribute to a single Wiener diffusion process with a diffusion constant of $\sigma$, which moves toward the correct decision boundary $b$. The drift rate of this combined process is calculated by adding the temporally constant input of the target-based process, with drift rate $\mu _{c}$, to the time-dependent input of the distractor-based process, with drift rate $\mu _{i}(t)$. The distractor-based process is modeled using a pulse-like gamma density function with a shape parameter of $\alpha$, which reaches its peak amplitude A at time $t_{peak}$ = ($\alpha$ - 1) $\times$ $\tau$, and then decreases back to zero. Reaction time (RT) is computed as the sum of the decision time required to reach the decision boundary b and a normally distributed non-decision (residual) time, with $\mu _{R}$ and $\sigma _{R}$. The model also accounts for starting point variability by sampling from a beta-shaped distribution $B$, which varies symmetrically around zero from $b_{1}$ to $b_{2}$. Importantly, the pulse-like distractor function allows DMC to produce both nDPs and pDPs because when the peak of the distractor-based function is reached relatively early, nDPs emerge, whereas when the peak is reached relatively late, pDPs arise.

In general, demonstrating that the model can reasonably fit the data would already indicate that a transient distractor activation architecture could account for various conflict tasks. Moreover, these model-based analyses are also helpful for more directly testing the transient distractor activation time course implemented by DMC. Specifically, DMC would predict that the parameter reflecting the time course of the distractor function should not meaningfully differ between duration conditions. Finally, comparing the other best-fitting parameters will generally help to better understand potential additional influences of stimulus duration on processing in these conflict tasks. For example, one might speculate that other processes could also be affected by presenting stimuli only for a short time rather than in a standard response-terminated way (e.g., target-based evidence accumulation as reflected in the controlled drift rate, response caution as reflected in decision boundaries, early sensory or late motor processes as reflected in non-decision times). As delta plot slopes can, in principle, also be affected by other processes (e.g., target-based drift rates, cf. Mittelstädt et al., 2023a), the generally similar slopes as a function of stimulus duration may, in principle, be also produced by subtle parameter changes across conditions. Thus, a more fine-grained investigation at a parameter level allows for a more precise examination of whether stimulus duration leaves different aspects of processing rather unaffected in conflict tasks, as has been found for non-conflict tasks (e.g., Ratcliff et al., 2016)

**Table 1:** Experiment 1. Best-fitting parameters of the diffusion model for conflict tasks (Ulrich et al., 2015) to the data of the four *Duration* (Long versus Short) $\times$ *Task* (Classic Simon versus Accessory Simon) conditions, as well as paired-samples t-tests comparing these estimates between the Long and Short conditions for both tasks

	Classic Simon					Acessory Simon				
	Estimate		Comparison			Estimate		Comparison		
	Short	Long	Short vs. Long			Short	Long	Short vs. Long		
Parameter	Mean (SE)	Mean (SE)	*t*(79)	*p*	*d*	Mean (SE)	Mean (SE)	*t*(79)	*p*	*d*
Amplitude *A* of distractor process	23.4 (1.32)	22.2 (1.14)	$-$0.95	.347	$-$0.10	14.2 (1.04)	13.5 (1.02)	$-$0.54	.589	$-$0.08
Peak time (ms) $\tau$ of distractor process	74.5 (7.79)	91.8 (8.62)	1.81	.074	0.23	145.2 (14.31)	177.5 (16.06)	1.42	.161	0.24
Decision boundary *b*	74.2 (2.49)	70.0 (2.35)	$-$1.93	.057	$-$0.19	66.6 (2.33)	62.8 (1.95)	$-$1.60	.113	$-$0.20
Drift rate $\mu _{c}$ of target process	0.6 (0.02)	0.6 (0.02)	0.82	.416	0.08	0.7 (0.02)	0.7 (0.02)	1.69	.095	0.19
Mean residual time $\mu _{R}$ (ms)	360.5 (5.06)	359.4 (4.36)	$-$0.35	.724	$-$0.02	351.3 (5.13)	354.0 (5.07)	0.73	.465	0.06
Variability of residual time (ms) $\sigma _{R}$	29.9 (1.95)	28.6 (1.76)	$-$0.56	.579	$-$0.08	33.7 (2.40)	32.6 (2.04)	$-$0.37	.716	$-$0.06
Shape $\alpha _{s}$ of starting point distribution	2.8 (0.07)	2.9 (0.08)	1.21	.228	0.18	2.9 (0.08)	3.0 (0.08)	1.47	.146	0.21
Goodness-of-fit (*RMSE*)	20.1 (1.11)	21.4 (1.07)	1.29	.200	0.13	17.7 (0.84)	19.5 (0.97)	1.86	.066	0.23

The fitting procedure used the R-Package DEoptim as implemented within the R-package DMCFun (Mackenzie & Dudschig, 2021). The step size was t $=$ 1 ms and the diffusion constant was fixed at $\sigma$ $=$ 4

**Fig. 2:**
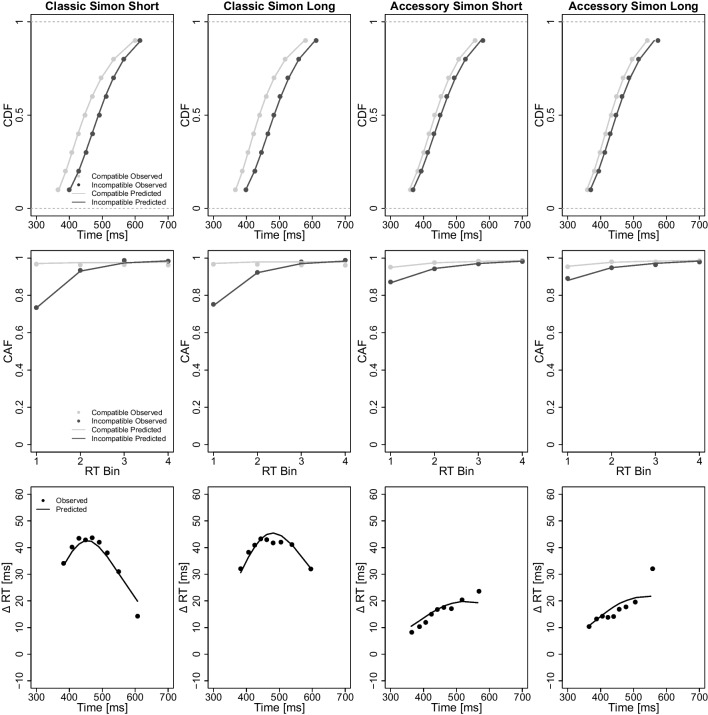
Experiment 1. Empirical results and DMC predictions of the best-fitting parameters. Each column represents one of the 2 (Classic/Accessory) $\times$ 2 (Long/Short) experimental conditions. The upper row depicts the cumulative distribution functions of correct RTs as a function of compatibility. The middle row depicts the conditional accuracy functions (CAFs) as a function of compatibility. The bottom row depicts RT delta plots

### Fitting Method

The DMC model was fitted to the individual data of the four conditions from each experiment with the R-package DMCfun (Mackenzie & Dudschig, 2021). We fitted the model simultaneously to condition-specific errors and reaction time (RT) distributions. This was achieved by minimizing the root-mean-squared error (RMSE) between observed and predicted values, a technique used in previous studies (e.g., Ellinghaus et al., 2018; Mittelstädt et al., 2022). Specifically, we used the DMCfun package to compute a cost value for both the percentile RT data ($RMSE_{RT}$) and error data ($RMSE_{CAF}$), and the total cost was a weighted sum of the two (for more details, see Mackenzie & Dudschig, 2021; Ulrich et al., 2015). In the fitting procedure, which employed a differential evolution algorithm (Mullen et al., 2011), the shape of the distractor process $\alpha$ was fixed at 2 for Experiments 1 and 2, but it was allowed to vary between the short and long condition.^5^ The remaining parameters of the model were always allowed to vary between the short and long condition; $b$ (decision boundary), $\tau$ (peak time of the automatic activation), $A$ (amplitude of the automatic process), $\mu _{c}$ (drift rate of the controlled process), $\mu _{R}$ (duration of the nondecisional processes) $\sigma _{R}$ (variability of the duration of nondecisional processes), and $\alpha _{s}$ (starting point variability of the superimposed accumulation process).

### Results

#### Experiment 1

The best-fitting DMC parameters as a function of *Task* and *Duration* are given in Table 1, and the corresponding visualizations of the model fits are given in Fig. 2. As can be seen from the low RMSE values and the visualization in Fig. 2, the model provides an excellent fit to the data across all conditions. These fits support the conclusion that models that incorporate the idea of transient, time-varying distractor-based activation are applicable across different stimulus durations. Interestingly, the peak time $$\tau $$ of the distractor process was numerically larger in the long than in the short condition for the classic Simon task, $$M = 91.80$$, 95% CI $$[74.64, 108.95]$$ versus $$M = 74.53$$, 95% CI $$[59.03, 90.03]$$, but this difference was only close to significance, $$F(1, 79) = 3.27$$, $$ MSE = 3,649.13$$, $$p =.074$$, $$\hat{\eta }^2_p =.040$$. For the accessory Simon task, the difference was also not significant, $$F(1, 79) = 2.00$$, $$ MSE = 20,851.08$$, $$p =.161$$, $$\hat{\eta }^2_p =.025$$. Of the remaining comparisons none were significant, either (see again Table 1).

#### Experiment 2

**Fig. 3:**
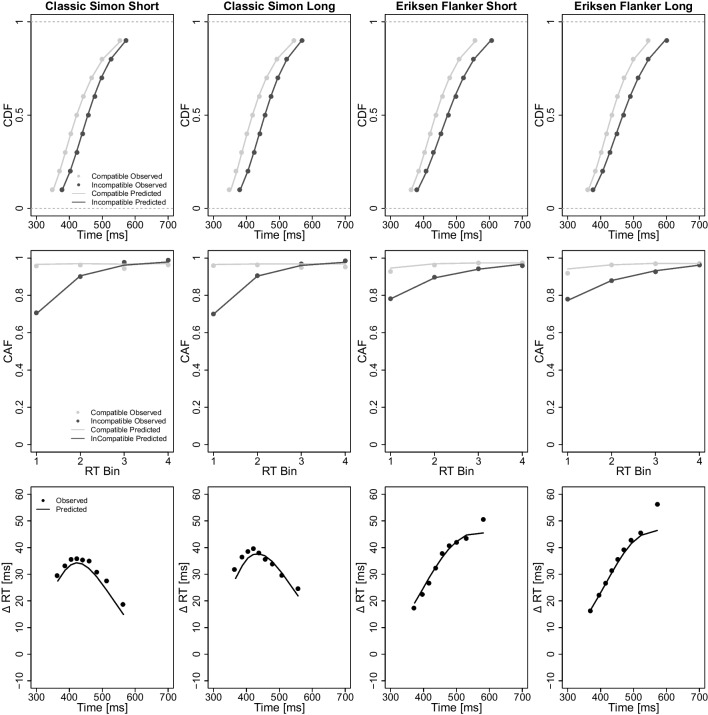
Experiment 2. Empirical results and DMC predictions of the best-fitting parameters. Each column represents one of the 2 (Simon/Flanker) $\times$ 2 (Long/Short) experimental conditions. The upper row depicts the cumulative distribution functions of correct RTs as a function of compatibility. The middle row depicts the conditional accuracy functions (CAFs) as a function of compatibility. The bottom row depicts RT delta plots

For Experiment 2, the best-fitting DMC parameters as a function of *Task* and *Duration* are given in Table 2, and the corresponding visualizations of the model fits are given in Fig. 3. Analogously to Experiment 1, visual inspection determined the fits to be very good. Furthermore, the peak time $$\tau $$ of the distractor process was only negligible larger in the long compared to the short condition, both for the Simon task, $$M = 64.75$$, 95% CI $$[51.53, 77.97]$$ versus $$M = 63.77$$, 95% CI $$[48.96, 78.59]$$, and for the Flanker task, $$M = 188.30$$, 95% CI $$[160.19, 216.42]$$ versus $$M = 176.07$$, 95% CI $$[148.31, 203.83]$$. Consistent with the DP analysis, neither for the Simon nor for the Flanker task, was this difference statistically significant, $$F(1, 80) = 0.01$$, $$ MSE = 3,563.02$$, $$p =.918$$, $$\hat{\eta }^2_p =.000$$ and $$F(1, 80) = 0.54$$, $$ MSE = 11,153.96$$, $$p =.463$$, $$\hat{\eta }^2_p =.007$$, respectively. Of the remaining comparisons none were significant, either (see again Table 2).

#### Experiment 3

**Fig. 4:**
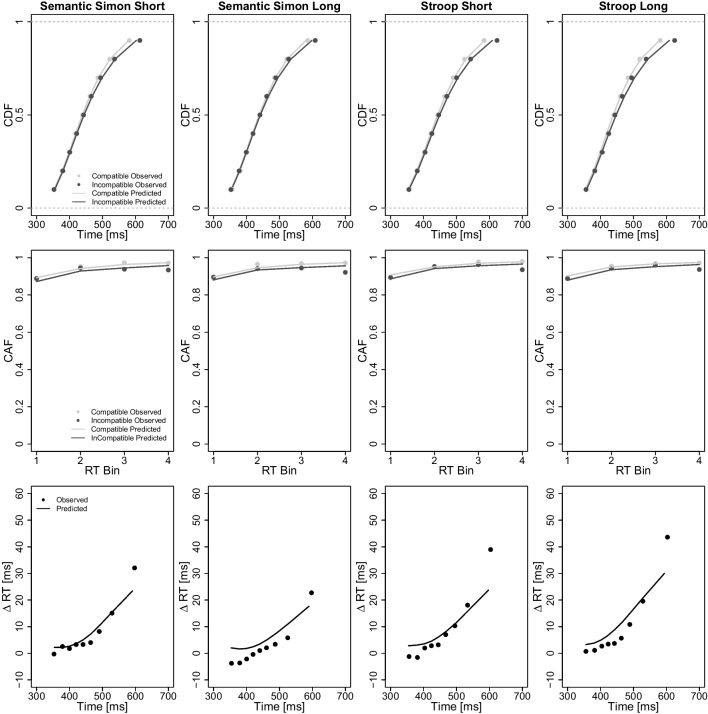
Experiment 3. Empirical results and DMC predictions of the best-fitting parameters. Each column represents one of the 2 (Simon/Stroop) $\times$ 2 (Long/Short) experimental conditions. The upper row depicts the cumulative distribution functions of correct RTs as a function of compatibility. The middle row depicts the conditional accuracy functions (CAFs) as a function of compatibility. The bottom row depicts RT delta plots

For Experiment 3, the best-fitting DMC parameters as a function of *Task* and *Duration* are given in Table 3, and the corresponding visualizations of the model fits are given in Fig. 4. As can be seen from the figure, the model once again reasonably fits the data. Consistent with the DP analysis, there were no significant differences for the $\tau$ of the distractor process between the short and long conditions, neither for the semantic Simon task nor for the Stroop task. Apart from slightly larger starting point variability $\alpha _{s}$ in the long condition compared to the short condition for the semantic Simon task, none of the remaining comparisons were significant (see again Table 3). Thus, even though in this experiment the shape of the distractor process $a$ was allowed to vary between conditions, there was no evidence that the shape was affected by stimulus duration.

#### Additional Modeling Analyses for Experiment 1–3

The DMC modeling analyses demonstrate that a modeling architecture incorporating the notion of transient rather than permanent distractor activation can reasonably account for the empirical data. Moreover, there was no evidence that the time course of distractor-based activation was affected by the duration conditions (i.e., parameter $\tau$ was not significantly different between the conditions). However, it might be possible that $\tau$ varied with other parameters when fitting the data to the specific conditions in such a way that blurs differences between conditions. Thus, as a further test to check for potential differences in $\tau$, we conducted additional fits for each experiment in which we only allowed $\tau$ to vary between conditions (i.e., conflict task X duration conditions), while all other parameters were not allowed to vary (for a similar approach, see Kelber et al., 2023). Critically, these additional fits also did not reveal any significant differences between $\tau$ within any conflict task (all *p* >.150). Thus, even when controlling for potential changes in other parameters between conditions (by estimating similar values), there was no evidence that the time course of distractor-based activation meaningfully differed between duration conditions.^6^

**Table 2:** Experiment 2. Best-fitting parameters of the diffusion model for conflict tasks (Ulrich et al., 2015) to the data of the four *Duration* (Long vs. Short) $\times$ *Task* (Classic Simon vs. Eriksen Flanker) conditions, as well as paired-samples t-tests comparing these estimates between the Long and Short conditions for both tasks

	Classic Simon					Eriksen Flanker				
	Estimate		Comparison			Estimate		Comparison		
	Short	Long	Short vs. Long			Short	Long	Short vs. Long		
Parameter	Mean (SE)	Mean (SE)	*t*(80)	*p*	*d*	Mean (SE)	Mean (SE)	*t*(80)	*p*	*d*
Amplitude *A* of distractor process	21.0 (1.14)	21.1 (1.20)	0.12	.907	0.01	20.1 (0.95)	21.3 (1.13)	1.09	.279	0.13
Peak time (ms) $\tau$ of distractor process	63.8 (7.45)	64.7 (6.64)	0.10	.918	0.02	176.1 (13.95)	188.3 (14.13)	0.74	.463	0.10
Decision boundary *b*	65.6 (2.21)	62.4 (1.89)	$-$1.49	.139	$-$0.17	62.1 (1.99)	57.9 (1.95)	$-$2.06	.043*	$-$0.24
Drift rate $\mu _{c}$ of target process	0.6 (0.02)	0.6 (0.02)	0.23	.815	0.02	0.6 (0.02)	0.6 (0.02)	0.26	.798	0.02
Mean residual time $\mu _{R}$ (ms)	347.5 (3.75)	349.2 (3.37)	0.65	.518	0.05	362.6 (4.27)	363.5 (4.33)	0.34	.736	0.02
Variability of residual time (ms) $\sigma _{R}$	27.7 (1.98)	26.0 (1.83)	$-$0.79	.430	$-$0.10	32.9 (2.47)	33.4 (2.03)	0.16	.871	0.02
Shape $\alpha _{s}$ of starting point distribution	2.7 (0.08)	2.8 (0.07)	1.01	.314	0.13	2.6 (0.07)	2.7 (0.08)	0.70	.486	0.08
Goodness-of-fit (*RMSE*)	21.5 (1.08)	21.9 (0.94)	0.40	.692	0.05	22.5 (1.09)	23.3 (1.22)	0.69	.489	0.07

The fitting procedure used the R-Package DEoptim as implemented within the R-package DMCFun (Mackenzie & Dudschig, 2021). The step size was t $=$ 1 ms and the diffusion constant was fixed at $\sigma$ $=$ 4

*Note: * Asterisk indicates significance at $p$ <.05

## General Discussion

In the present study, we investigated the influence of stimulus (target and distractor) duration on processing across several conflict tasks with manual responses. We were particularly interested if distractor-based activation in conflict tasks is more accurately approximated by transient or by permanent activation architectures and employed distributional (delta plot) analyses to investigate this issue. While permanent activation accounts predict DPs to turn more negative with short (as compared to response-terminated) stimulus duration, transient activation accounts predict the DPs slopes to be largely unaffected by stimulus duration. Overall, the results of three Experiments (N $=$ 242) provide evidence for the validity of transient rather than permanent activation models. Specifically, the delta plot slopes were virtually unaffected by stimulus duration across all tasks (classic Simon task, accessory Simon task, word-based Simon task, Eriksen flanker task, Stroop task). The only exception to this pattern was the classic Simon task in Experiment 1. Here, stimulus duration exerted a positive influence on DP slopes, as predicted by permanent activation architectures. Nevertheless, this result pattern was not replicated in Experiment 2. Accordingly, the behavioral results suggest that transient activation models are a more viable account of distractor-based activation in the employed conflict tasks than permanent activation models.

**Table 3:** Experiment 3. Best-fitting parameters of the diffusion model for conflict tasks (Ulrich et al., 2015) to the data of the four *Duration* (Long vs. Short) $\times$ *Task* (Semantic Simon vs. Stroop) conditions, as well as paired-samples t-tests comparing these estimates between the Long and Short conditions for both tasks

	Semantic Simon					Stroop				
	Estimate		Comparison			Estimate		Comparison		
	Short	Long	Short vs. Long			Short	Long	Short vs. Long		
Parameter	Mean (SE)	Mean (SE)	*t*(80)	*p*	*d*	Mean (SE)	Mean (SE)	*t*(80)	*p*	*d*
Amplitude *A* of distractor process	22.1 (2.02)	24.0 (2.14)	0.80	.428	0.10	22.3 (1.92)	20.6 (1.83)	$-$0.73	.468	$-$0.10
Peak time (ms) $\tau$ of distractor process	215.1 (15.09)	231.4 (15.27)	0.80	.427	0.12	211.5 (16.87)	237.9 (15.77)	1.26	.210	0.18
Decision boundary *b*	62.9 (1.77)	64.4 (2.24)	0.81	.423	0.09	68.8 (2.25)	65.8 (2.12)	$-$1.48	.142	$-$0.15
Drift rate $\mu _{c}$ of target process	0.5 (0.02)	0.5 (0.02)	1.10	.276	0.10	0.6 (0.02)	0.5 (0.02)	$-$1.47	.146	$-$0.13
Mean residual time $\mu _{R}$ (ms)	338.1 (6.08)	334.1 (5.78)	$-$1.14	.258	$-$0.07	336.1 (5.49)	334.7 (4.62)	$-$0.39	.697	$-$0.03
Variability of residual time (ms) $\sigma _{R}$	29.8 (2.59)	26.9 (2.43)	$-$1.06	.293	$-$0.13	30.5 (2.46)	27.1 (2.26)	$-$1.11	.269	$-$0.16
Shape $\alpha _{s}$ of starting point distribution	3.0 (0.09)	3.2 (0.09)	2.03	.045*	0.21	2.9 (0.09)	3.0 (0.09)	0.79	.429	0.11
Shape *a* of the gamma distribution	2.1 (0.09)	2.1 (0.09)	$-$0.13	.898	$-$0.02	2.1 (0.09)	2.2 (0.08)	1.08	.286	0.15
Goodness-of-fit (*RMSE*)	28.4 (1.31)	27.9 (1.29)	$-$0.38	.703	$-$0.04	25.8 (1.28)	26.6 (1.19)	0.60	.550	0.07

The fitting procedure used the R-Package DEoptim as implemented within the R-package DMCFun (Mackenzie & Dudschig, 2021). The step size was t $=$ 1 ms and the diffusion constant was fixed at $\sigma$ $=$ 4

*Note: * Asterisk indicates significance at $p$ <.05

Our model-based analysis was consistent with this interpretation. First, the model fits demonstrate that the DMC model, which incorporates the notion of transient, time-varying distractor-based activation, can provide a good fit to the data across all conditions. This provides further support for the idea that transient activation accounts approximate the cognitive mechanisms underlying a range of conflict tasks relatively well. Second, the $\tau$ parameter of the DMC model which reflects the time course of distractor activation was virtually unaffected by stimulus duration across these tasks. Moreover, the model-based analyses also do not provide evidence that any other processes (reflected in model parameters) are meaningfully affected by stimulus duration, which seems in line with analyses using the standard diffusion model in non-conflict tasks (cf. Ratcliff et al., 2016). Moreover, the model-based analyses also do not suggest that any other processes (i.e., parameters) are meaningfully affected by stimulus duration, which seems in line with analyses employing the standard diffusion model in non-conflict tasks (Ratcliff et al., 2016). The only exception was that decision boundaries were significantly larger in the short compared to the long condition for the Eriksen flanker task in Experiment 2, and although not significant, a similar descriptive difference was observed for the other conflict tasks in Experiments 1 and 2. As mean RTs were, on average, significantly slower in the short than long condition in Experiment 1 (5 ms) and Experiment 2 (3 ms), one might speculate that this RT slowdown is due to participants being more cautious (and hence raising bounds) when the stimulus only briefly appears.

Obviously, we can in principle not rule out that effects of stimulus duration on delta plots maybe even smaller than those considered with the present sample sizes (cf. sample size justification section), and hence may require even larger sample sizes to be detected with sufficient statistical power. However, considering the rather consistent result pattern across three experiments, as well as the coherent modeling results, we suggest that the present study refute the predictions of permanent activation models which assume the distractor to continuously input into the automatic process (Cohen et al., 1990; Logan, 1980). Rather, the observed pattern of results is consistent with transient activation architectures assuming a brief and pulse-like activation that is virtually unaffected by stimulus duration (Ellinghaus et al., 2018; Nishimura & Yokosawa, 2010, Experiment 1; Ulrich et al., 2015; Usher & McClelland, 2001; Wühr & Heuer, 2017; Wühr & Heuer, 2018). Crucially, our study extends these findings of previous studies by examining different types of distractors and tasks employing analyses based on delta functions and parameter estimation. In that sense, our results extend previous findings suggesting that different conflict tasks that differ with regard to their distributional properties might still be captured by the same underlying mechanism such as proposed by DMC (Luo & Proctor, 2020).

More broadly, the present results are in line with the idea that humans can rapidly exploit spatial information for action planning based on current task demands (Logan, 1978, 1989; Neumann, 1984). For instance, the idea that visual information can directly affect movement preparation dependent on the agent’s current intentions or task demands not only has a long tradition in experimental psychology (Münsterberg, 1889; Woodworth, 1937), but also received a lot of support with modern techniques in the context of masked priming studies (Fehrer & Raab, 1962; for an overview, see Kouider & Dehaene, 2007). One of the main conclusions from this field of research is that response activation by visual stimuli does not depend on conscious awareness of the stimulus (Vorberg et al., 2003). In turn, future studies might employ masking also in conflict tasks, for example, to address the question of whether transient activation is invariant against the degradation of the corresponding visual information held in iconic memory (cf. Ellinghaus & Miller, 2018). For example, assuming that a given conflict effect primarily reflects relatively direct motor activation via the dorsal route (cf., Milner & Goodale, 2006), one could expect to find a dissociation between stimulus visibility and conflict effects. However, potential contributions of distractor information based on perceptual or semantic analysis (e.g., in the Eriksen flanker or Stroop task) might be more sensitive to masking.

Finally, it should be noted that the present Experiments suggest that a transient activation architecture generally underlies conflict tasks $within$ the visual modality. A logical next step would be to investigate generalization also $across$ different modalities. So far, the literature seems relatively inconclusive in this regard. For example, when using auditory distractors in an accessory Simon task with visual targets, Nishimura and Yokosawa (2010) (Experiment 2) observed a compatibility effect only when the distractor appeared but not when it disappeared on either side at the time of target presentation, although both onsets and offsets were effective when distractors and targets were visual (Experiment 1). Furthermore, although Xiong and Proctor (2016) report the auditory Simon effect to decrease across bins, more often increasing effects with auditory stimuli have been reported (Proctor & Shao, 2010; Wascher et al., 2001). In light of these findings, it is well conceivable that visual stimuli in particular cause a transient activation while auditory stimuli may input into the response selection process rather permanently. Furthermore, it remains to be seen to what extent the present findings generalize to other output modalities (e.g., vocal responses).

To conclude, the results we report here support the view that the nature of distractor activation in conflict tasks is more accurately approximated by transient than by permanent activation architectures. Crucially, the present work makes the theoretically important step to generalize this conclusion across multiple tasks within the visual modality. In the future, it will be interesting to search for potential anomalies and inconsistencies from this established pattern, so that existing theories can be extended or refined.

## Figures and Tables

**Fig. 5:**
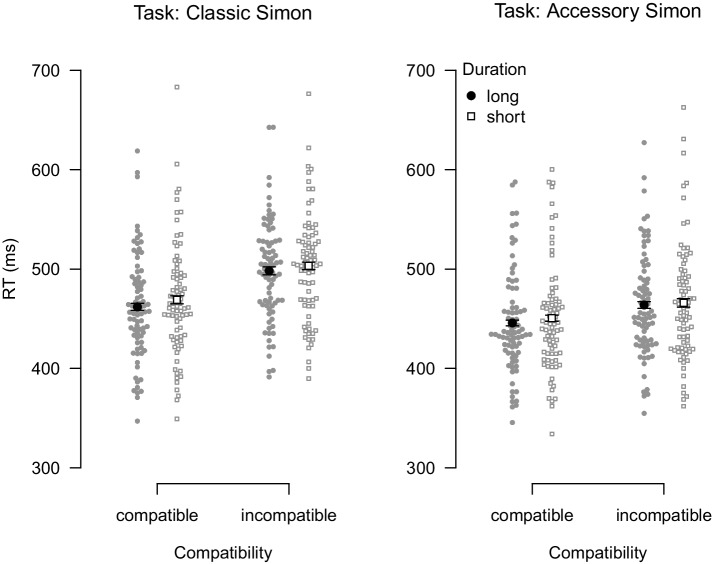
Experiment 1. RT as a function of *Compatibility* and *Duration*, respectively for the classic Simon task (left) and the accessory Simon task (right). Error bars reflect 95% CIs after Morey et al. (2008)

**Fig. 6:**
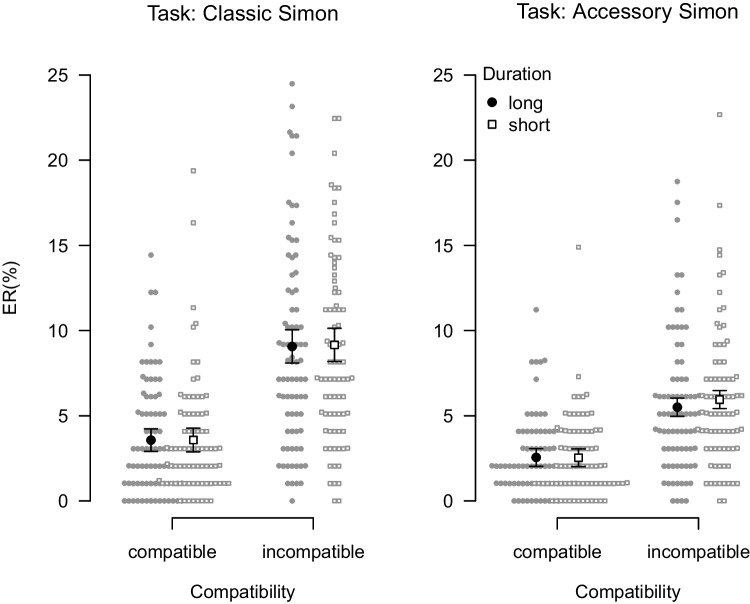
Experiment 1. ER as a function of *Compatibility* and *Duration*, respectively for the classic Simon task (left) and the accessory Simon task (right). Error bars reflect 95% CIs after Morey et al. (2008)

**Fig. 7:**
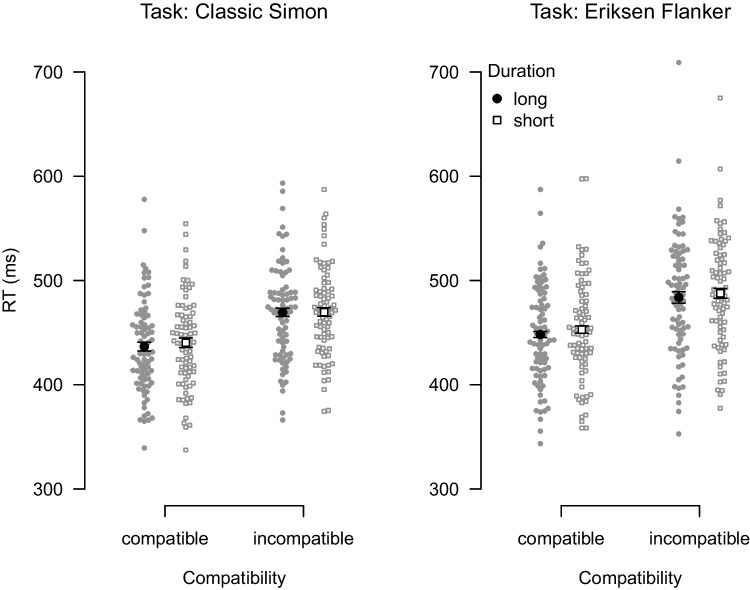
Experiment 2. RT as a function of *Compatibility* and *Duration*, respectively for the classic Simon task (left) and the Eriksen flanker task (right). Error bars reflect 95% CIs after Morey et al. (2008)

**Fig. 8:**
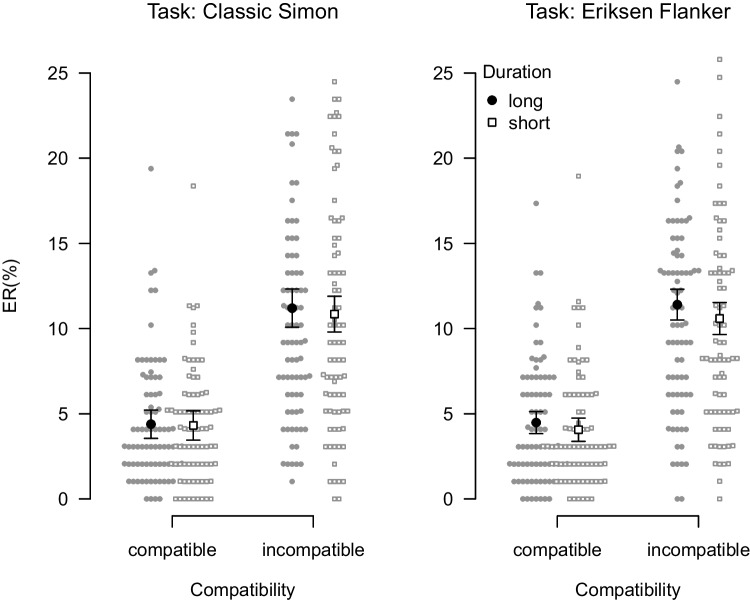
Experiment 2. ER as a function of *Compatibility* and *Duration*, respectively for the classic Simon task (left) and the Eriksen flanker task (right). Error bars reflect 95% CIs after Morey et al. (2008)

**Fig. 9:**
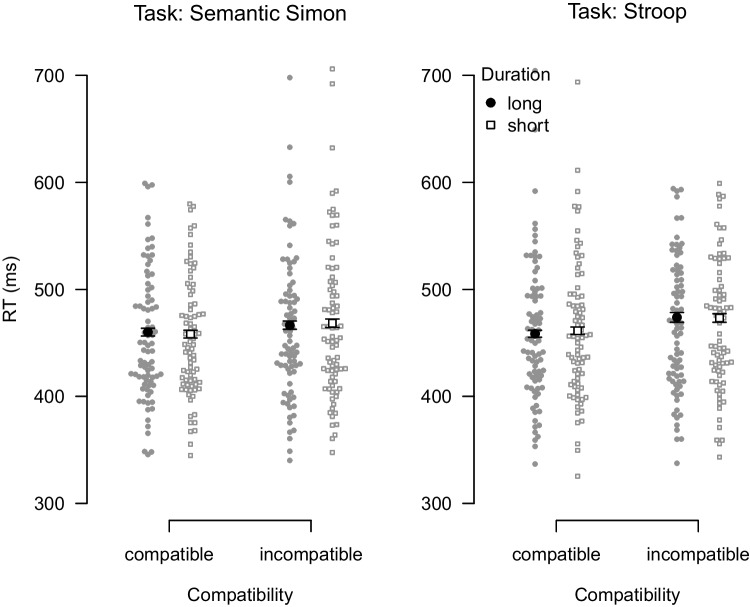
Experiment 3. RT as a function of *Compatibility* and *Duration*, respectively for the semantic Simon task (left) and the Stroop task (right). Error bars reflect 95% CIs after Morey et al. (2008)

**Fig. 10:**
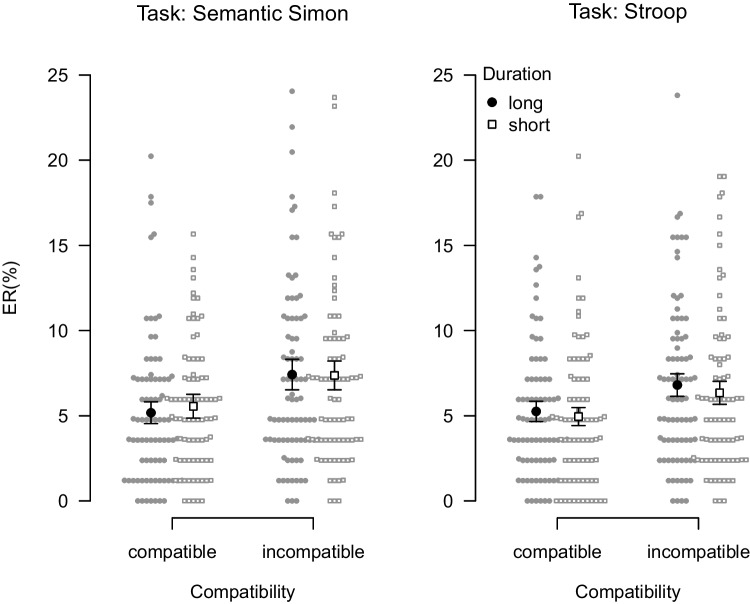
Experiment 3. ER as a function of *Compatibility* and *Duration*, respectively for the semantic Simon task (left) and the Stroop task (right). Error bars reflect 95% CIs after Morey et al. (2008)

## Data Availability

Open Practice Statement and availability of data and materials: Raw data of all experiments are available via the Open Science Framework (OSF) at https://osf.io/cb36q/. Preregistrations of Experiment 1, Experiment 2, and Experiment 3 is available via the OSF at https://osf.io/bdftk, https://osf.io/d62mv and https://osf.io/n2vaw, respectively.

## Appendix

### Mean Results

#### Experiment 1

The RT data of Experiment 1 are given in Fig. 5. In the corresponding ANOVA, the main effect of *Compatibility* reached significance, $$F(1, 79) = 301.40$$, $$ MSE = 358.51$$, $$p <.001$$, $$\hat{\eta }^2_p =.792$$, reflecting faster reactions in compatible trials ($$M = 457\ ms$$) compared to incompatible trials ($$M = 483\ ms$$). The main effect of *Duration* was also significant, $$F(1, 79) = 13.42$$, $$ MSE = 270.94$$, $$p <.001$$, $$\hat{\eta }^2_p =.145$$, reflecting slower reactions with the short compared to the long stimulus presentation, namely $$M = 472\ ms$$ vs. $$M = 467\ ms$$, respectively. The main effect of *Task* was significant, too, $$F(1, 79) = 167.32$$, $$ MSE = 674.17$$, $$p <.001$$, $$\hat{\eta }^2_p =.679$$, reflecting slower reactions for the classic compared to the accessory Simon task, namely $$M = 483\ ms$$ vs $$M = 457\ ms$$, respectively. The interaction of *Task* and *Compatibility* was significant, $$F(1, 79) = 47.09$$, $$ MSE = 289.16$$, $$p <.001$$, $$\hat{\eta }^2_p =.373$$, reflecting a larger Simon effect for the classic as compared to the accessory task, namely $$M = 35.2\ ms$$ vs $$M = 16.8\ ms$$, respectively. Of the remaining effects, none were significant (all $$F$$ $$\le $$ 2.25, and all $$p$$ $$\ge $$.138).

The ER data of Experiment 1 are given in Fig. 6. In the corresponding ANOVA, the main effect of *Compatibility* reached significance, $$F(1, 79) = 108.03$$, $$ MSE = 28.17$$, $$p <.001$$, $$\hat{\eta }^2_p =.578$$, reflecting less errors in compatible trials ($$M = 3.06\ \%$$) compared to incompatible trials ($$M = 7.42\ \%$$). The main effect of *Task* was significant, too, $$F(1, 79) = 83.10$$, $$ MSE = 9.38$$, $$p <.001$$, $$\hat{\eta }^2_p =.513$$, reflecting more errors for the classic compared to the accessory Simon task, namely $$M = 6.34\ \%$$ vs $$M = 4.14\ \%$$, respectively. The interaction of *Task* and *Compatibility* was significant, $$F(1, 79) = 19.99$$, $$ MSE = 11.15$$, $$p <.001$$, $$\hat{\eta }^2_p =.202$$, reflecting a larger Simon effect for the classic as compared to the accessory task, namely $$M = 5.54\ \%$$ vs $$M = 3.18\ \%$$, respectively. Of the remaining effects, none were significant (all $$F$$ $$\le $$ 0.61, and all $$p$$ $$\ge $$.437).

#### Experiment 2

The RT data of Experiment 2 are given in Fig. 7. In the corresponding ANOVA, the main effect of *Compatibility* reached significance, $$F(1, 80) = 503.97$$, $$ MSE = 353.07$$, $$p <.001$$, $$\hat{\eta }^2_p =.863$$, reflecting faster reactions in compatible trials ($$M = 444\ ms$$) compared to incompatible trials ($$M = 478\ ms$$). The main effect of *Duration* was also significant, $$F(1, 80) = 9.08$$, $$ MSE = 177.16$$, $$p =.003$$, $$\hat{\eta }^2_p =.102$$, reflecting slower reactions with the short compared to the long stimulus presentation, namely $$M = 463\ ms$$ vs $$M = 459\ ms$$, respectively. The main effect of *Task* was significant, too, $$F(1, 80) = 21.32$$, $$ MSE = 1,513.41$$, $$p <.001$$, $$\hat{\eta }^2_p =.210$$, reflecting faster reactions for the Simon compared to the Flanker task, namely $$M = 454\ ms$$ vs $$M = 468\ ms$$, respectively. Of the remaining effects, none were significant (all $$F$$ $$\le $$ 2.95, and all $$p$$ $$\ge $$.090).

The ER data of Experiment 2 are given in Fig. 8. In the corresponding ANOVA, the main effect of *Compatibility* reached significance, $$F(1, 80) = 162.96$$, $$ MSE = 44.62$$, $$p <.001$$, $$\hat{\eta }^2_p =.671$$, reflecting less errors in compatible trials ($$M = 4.31\ \%$$) compared to incompatible trials ($$M = 11.01\ \%$$). Of the remaining effects, none were significant (all $$F$$ $$\le $$ 2.72, and all $$p$$ $$\ge $$.103).

#### Experiment 3

The RT data of Experiment 3 are given in Fig. 9. In the corresponding ANOVA, the main effect of *Compatibility* reached significance, $$F(1, 80) = 33.36$$, $$ MSE = 583.36$$, $$p <.001$$, $$\hat{\eta }^2_p =.294$$, reflecting faster reactions in compatible trials ($$M = 460\ ms$$) compared to incompatible trials ($$M = 470\ ms$$). The main effect of *Task* was significant, too, $$F(1, 80) = 4.47$$, $$ MSE = 439.69$$, $$p =.038$$, $$\hat{\eta }^2_p =.053$$, reflecting faster reactions for the Simon compared to the Stroop task, namely $$M = 463\ ms$$ vs $$M = 467\ ms$$, respectively. The interaction of *Task* and *Compatibility* was significant, $$F(1, 79) = 47.09$$, $$ MSE = 289.16$$, $$p <.001$$, $$\hat{\eta }^2_p =.373$$, reflecting a larger effect for the Stroop as compared to the Simon task, namely $$M = 13.6\ ms$$ vs $$M = 8.3\ ms$$, respectively. Of the remaining effects, none were significant (all $$F$$ $$\le $$ 3.36, and all $$p$$ $$\ge $$.071).

The ER data of Experiment 3 are given in Fig. 10. In the corresponding ANOVA, the main effect of *Compatibility* reached significance, $$F(1, 80) = 29.53$$, $$ MSE = 16.83$$, $$p <.001$$, $$\hat{\eta }^2_p =.270$$, reflecting less errors in compatible trials ($$M = 5.23\ \%$$) compared to incompatible trials ($$M = 6.98\ \%$$). Of the remaining effects, none were significant (all $$F$$ $$\le $$ 2.11, and all $$p$$ $$\ge $$.150).

## Footnotes

1 It is worth noting that Ellinghaus et al. (2018) also used the diffusion model for conflict tasks (DMC) to fit the aggregated data of their experiments. However, they fixed all parameters except for the $\tau$ parameter reflecting the automatic activation time course, leaving it unclear whether a combination of changes in tau with different parameters could have produced the observed pattern. For example, Mittelstädt and Miller (2020) found similar delta plot slopes while varying different parameters in the DMC. Finally, it should be noted that Ellinghaus et al. (2018) raised some concerns regarding the interpretation of their empirical delta plot results, given the relatively small sample size (N $=$ 20) in their study. Specifically, they cautioned that the statistical power may have been insufficient to detect subtle differences in the delta plot slopes between conditions and that larger sample sizes might be needed to confirm the robustness of the observed effects.

2 According to our preregistration plan, we originally intended to collect data from a sample of 80 participants in each experiment. However, due to an unintentional extended period of time during which the experiment link remained active, some additional participants were recruited beyond the original target sample size. Nonetheless, we found that the results of both experiments were highly consistent and comparable when analyzed solely based on the data of the initial 80 participants.

3 While online experiments naturally lack the same level of control over stimulus presentation timing as their laboratory counterparts, it has been shown that internet-based mental chronometry studies produce performance measures comparable to those collected in the laboratory (Miller et al., 2018).

4 As the delta plots in the Simon tasks were initially increasing and then decreasing, we additionally only fitted the regression lines to the later percentiles where the delta plots were decreasing. Comparing the slopes obtained from these fits revealed similar results to the ones reported in the main text.

5 Initially, we also fixed $\alpha$ when fitting the data of Experiment 3. However, the banana-like increasing time course of the Stroop task was not adequately captured, suggesting that the typically used shape of $\alpha$ = 2 is not adequate. Thus, we decided to also vary $\alpha$ in this experiment. Note that rerunning the fits of Experiments 1 and 2 while also varying $\alpha$ between conditions revealed similar results.

6 The specific comparisons of the best-fitting tau parameters between the task-specific short and long conditions of each experiment and the corresponding visualizations of the model fits are provided via OSF at https://osf.io/cb36q/. As can be seen, even though only $\tau$ was allowed to vary, the model still fitted the data reasonably well.
